# Supplementary material for: Hippocampal area CA2 activity supports social investigation following an acute social stress
Source: Mol Psychiatry. 2024 Nov 15;30(6):2284–96. doi: 10.1038/s41380-024-02834-9 (PMC12092241; doi:10.1038/s41380-024-02834-9)
Supplement: Supplementary file 1 — Radzicki et al, Supplementary Figures [file 41380_2024_2834_MOESM1_ESM.docx]

**Hippocampal area CA2 activity supports social investigation following an acute social stress_Supplemental data**

Daniel Radzicki^1^, Katharine E. McCann^1, 2^, Georgia M. Alexander^1^, Serena M. Dudek^1^ *

^1^ *Neurobiology Laboratory, National Institute of Environmental Health Sciences, National Institute of Health, Research Triangle Park, North Carolina, 27713, USA*

^2^ *Current address: Neuroscience Undergraduate Program and School of Psychology, Georgia Institute of Technology, Atlanta, Georgia, 30332, USA*

^*^ Corresponding author

**
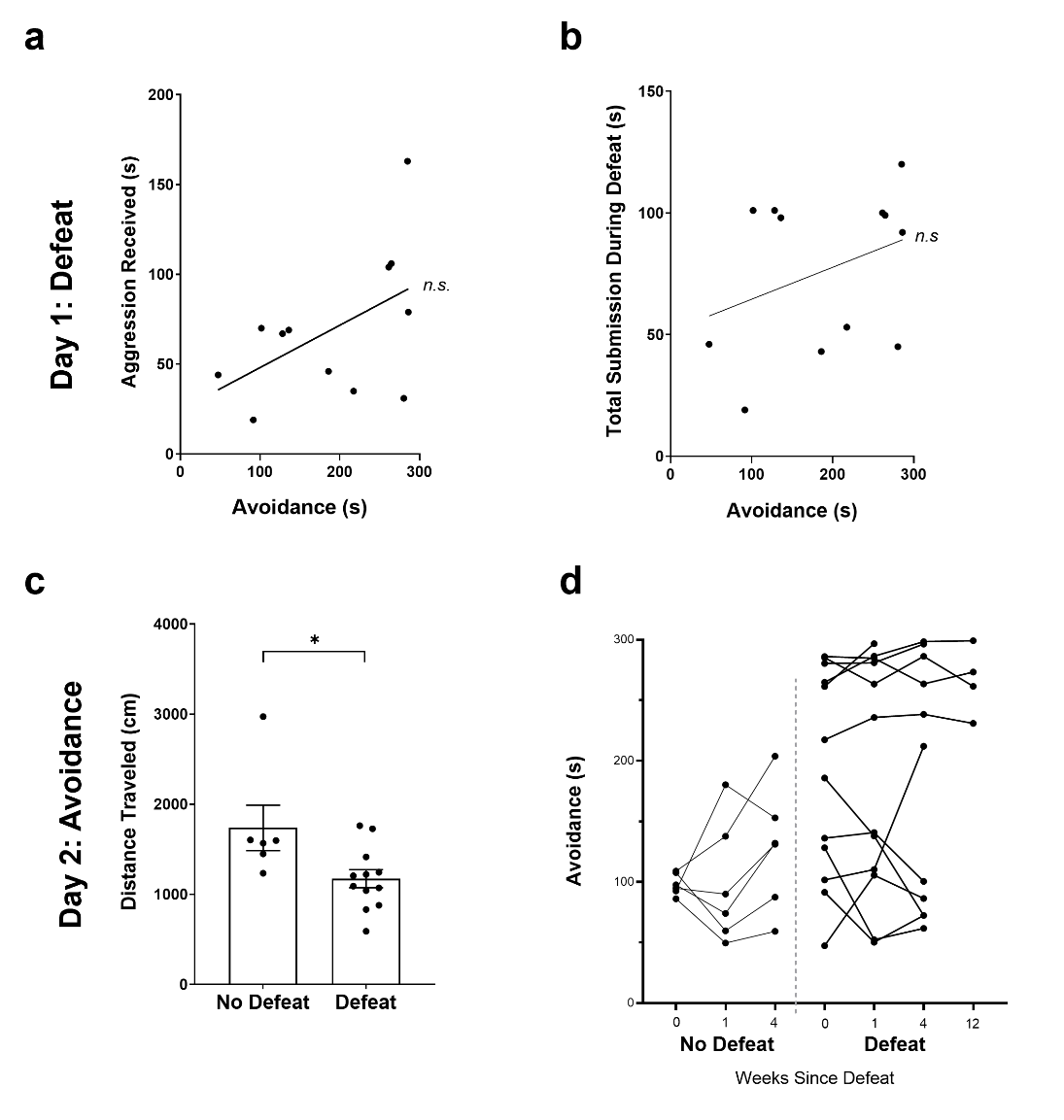
**

**Supplementary Figure 1**: Comparison of subject mouse behavioral metrics during day 1 defeat and day 2 avoidance test.

**a.** No significant correlation was observed between the amount of aggression a subject mouse received during the defeat and the amount of avoidance observed 24 hours after defeat (N=12 defeated mice, R^2^=0.2573, *p*>0.05). **b.** No significant correlation was observed between total submissive behavior exhibited by subject mice during defeat and the amount of avoidance observed 24 hours after defeat (N=12 defeated mice, R^2^=0.2727, *p*>0.05). **c.** On average, defeated C57BL/6J mice explored the day 2 arena significantly less than the non-defeated controls during the five-minute avoidance test (No Defeat: 1737.81 ± 253.3 cm vs. Defeat: 1173 ± 99.2 cm, N=6 & 12 mice, respectively, unpaired *t-test* *p*=0.02). **d.** Highly avoidant, defeated mice (>200 seconds spent in the far zone) remained avoidant for up to three months following a single bout of aSD when exposed to a littermate of the aggressive CD1 when compared to the more investigative, defeated mice (week 0, Defeat-Avoidant: 265.84 ± 10.6 second vs. week 0, Defeat-Investigative: 115.14 ± 19.1 second, N=6 & 6, unpaired *t-test* p<0.0001). * *p<*0.05.

**
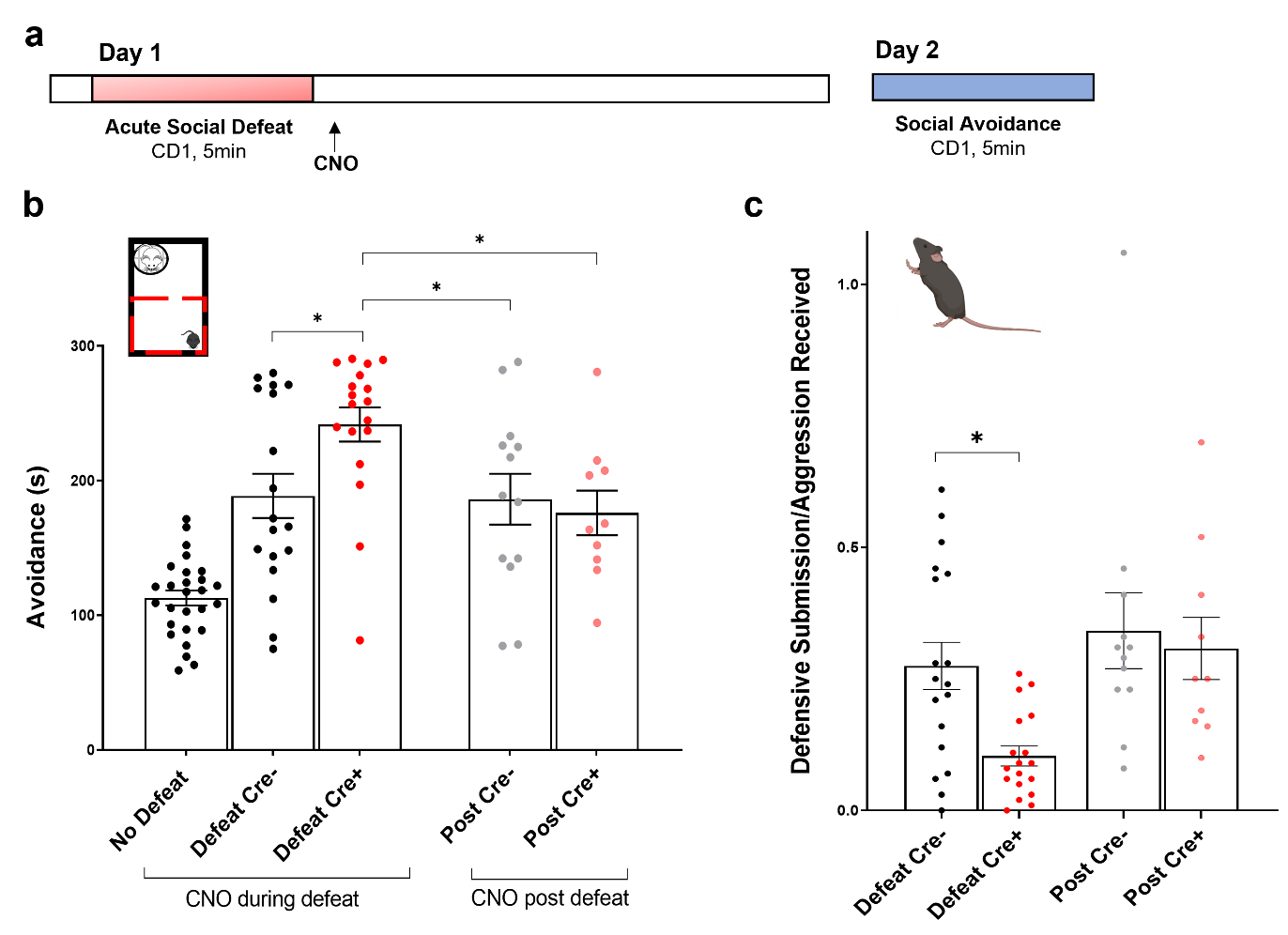
**

**Supplementary Figure 2**: Chemogenetic inactivation of rCA2 after the defeat has no effect on subject avoidance 24 hours post-defeat.

**a.** Acute social defeat experimental timeline. Five mg/kg of clozapine-N-oxide (CNO) was delivered on day 1, immediately following defeat by a CD1 aggressor mouse via IP injection in Amigo2-icreERT2 male mice (16-19wks of age) that either did (Cre+) or did not (Cre-) express cre-recombinase in area CA2 neurons. Twenty-four hours after defeat, day 2, mice were tested for avoidance/investigation in a novel context containing a littermate of the CD1 aggressor mouse. **b.** Inhibition of area CA2, via the activation of a virally expressed Gi-DREADD construct, immediately following aSD resulted in an avoidance phenotype similar to that of Defeat Cre- animals that received CNO prior to the defeat at 24 hours and significantly lower than Cre+ mice wherein CA2 activity was inhibited during the defeat (data shown are replotted from Figure 2 with additional ‘No Defeat’ animals; Defeat Cre+: 241.64 ± 12.7 seconds vs. Post Cre-: 186.21 ± 18.9 seconds vs. Post Cre+: 176.01 ± 16.6 seconds, N=18, 13, 10, respectively, one-way ANOVA F=16.41 p<0.0001; Defeat Cre+ vs. Post Cre-, Holm-Sidak multiple comparisons test *p*=0.023, Defeat Cre+ vs. Post Cre+, Holm-Sidak *p*=0.016). **c.** Confirmation that the subject defensive behavior during the defeat was similar in mice given CNO after the defeat and cre- mice given prior to defeat. Defensive submission values, normalized to the amount of aggression received, were equivalent to those of defeated Cre- mice that received CNO prior to defeat (Defeat Cre-: 0.28 ± 0.04 vs. Defeat Cre+: 0.10 ± 0.04 vs. Post Cre-: 0.34 ± 0.07 vs. Post Cre+: 0.31 ± 0.06, N=18, 18, 12, 10 mice, respectively, one-way ANOVA F=5.52 *p*=0.0022; Defeat Cre- vs. Defeat Cre+, Holm-Sidak *p*=0.026, Defeat Cre+ vs. Post Cre-, Holm-Sidak *p*=0.004, Defeat Cre+ vs. Post Cre+, Holm-Sidak *p*=0.026). * *p<*0.05, ** *p*<0.01.

**
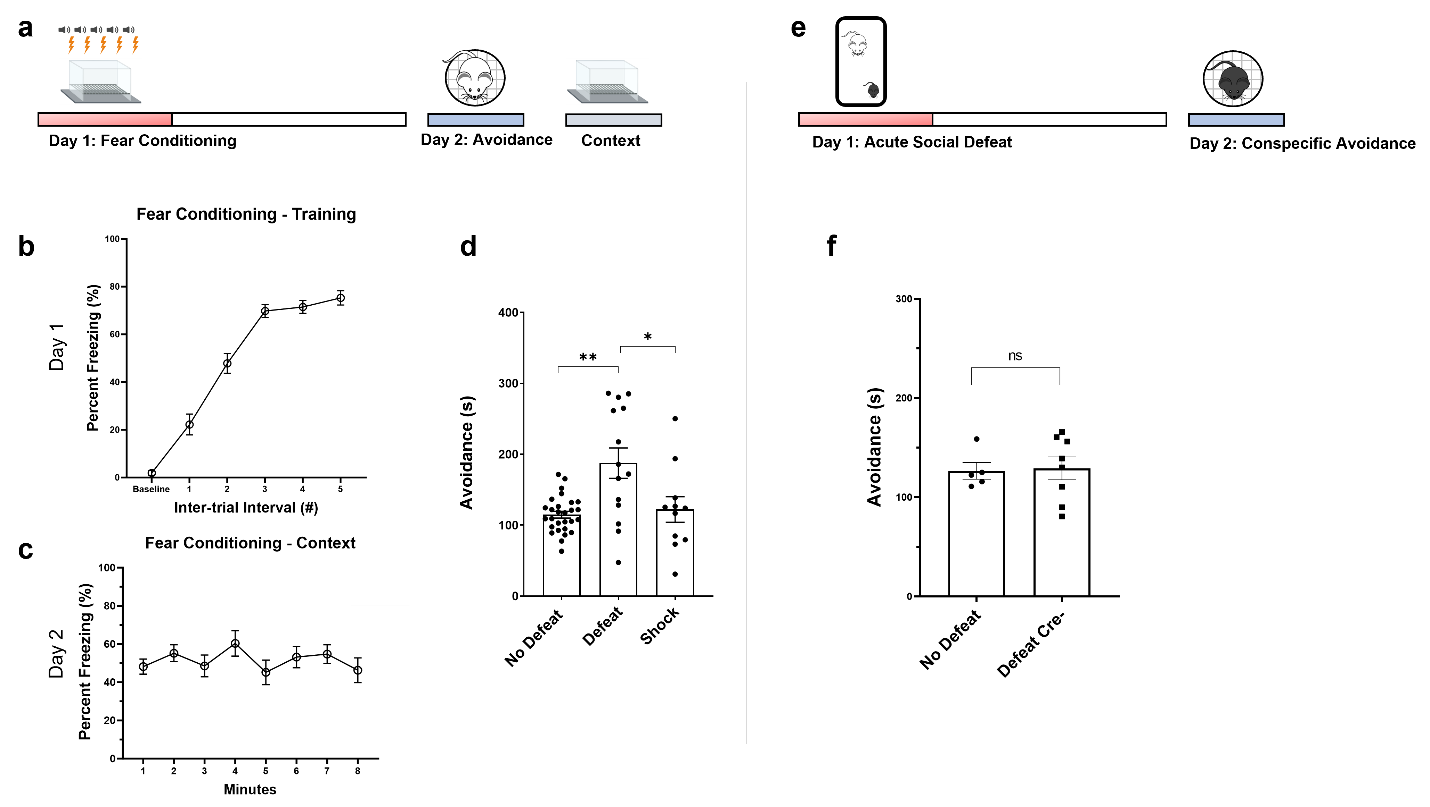
**

**Supplementary Figure 3**: Avoidance is specific to the acute defeat stressor.

**a.** Experimental timeline for a subset of animals in which the aSD stressor was replaced with a fear conditioning protocol involving five tone-shock pairings on day 1, followed by a day 2 avoidance test and screening for the acquisition of contextual fear. **b.** Wildtype mice showed a graded increase percent time spent freezing during the administration of five 0.6 mA footshocks at 30 second intervals (interevent interval 1: 22.2 ± 4.4 % freezing vs. interval 5: 75.3 ± 3.0%, N=11 mice tested). **c.** Twenty-four hours later, mice that underwent fear conditioning continued to exhibit freezing behavior when placed back in the day 1 context (minute 1: 48.2 ± 4.0% versus minute 8: 46.3 ± 6.5%, N=11 total mice tested). **d.** Twenty-four hours after the administration of a non-social stressor (i.e. footshocks), shock conditioned mice spent a comparable amount of time in the far zone as the no defeat control animals and significantly less time than wild type, defeated mice (No Defeat controls: 114.84 ± 4.8 seconds vs. Defeat: 190.49 ± 25.0 seconds vs. Shock Conditioned: 122.08 ± 18.0 seconds, N=28, 12, 11 mice, respectively one-way ANOVA F=9.47 p=0.0003; No Defeat vs. Defeat, Holm-Sidak multiple comparisons test *p*=0.0003; Defeat vs. Shock Conditioned, Holm-Sidak *p*=0.0063). **e.** Experimental timeline for a subset of animals that were socially defeated by a CD1 aggressor mouse on day 1 and then allowed to investigate a novel, age matched male conspecific (C57BL/6J) on day 2. **f.** When compared to non-defeat control mice, socially defeated mice spent an equivalent amount of time in the far zone of a novel arena when allowed to interact with a novel, conspecific 24 hours after being defeated by a CD1 aggressor (No Defeat: 126.70 ± 8.4 seconds spent in the far zone vs. Defeat Cre-: 129.19 ± 11.5, N=5, 8 mice, respectively, unpaired *t-test p*=0.88). * *p<*0.05, ** *p*<0.01.


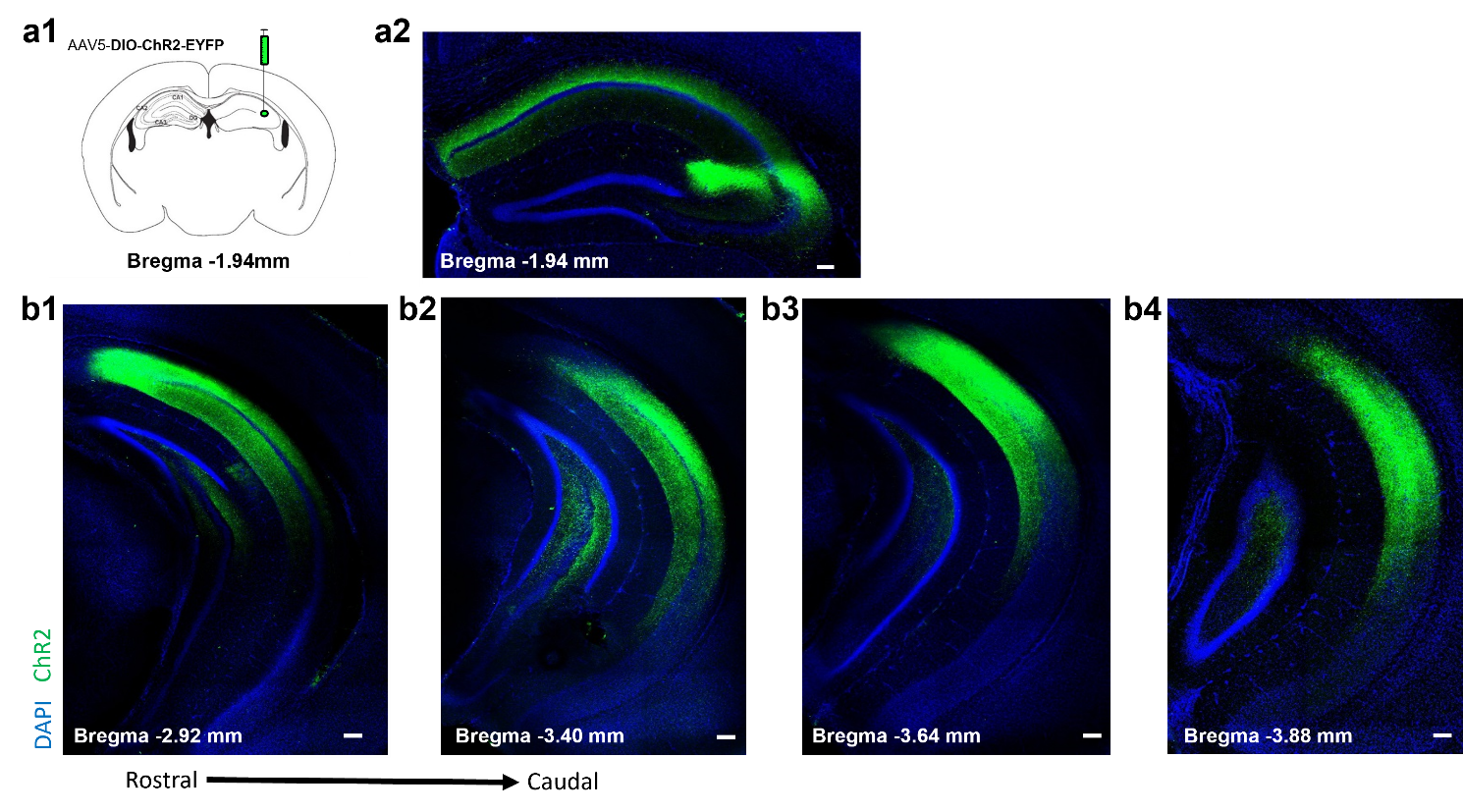


**Supplementary Figure 4:** Rostral CA2 axons target the caudal hippocampus in cre+ Amigo2-icreERT2 mice.

**a1.** Intracranial injection of 500 µl of a cre-recombinase dependent virus containing channelrhodopsin and an EYFP tag were delivered into the rostrodorsal CA2 (rCA2) of an Amigo2-icreERT2 mouse. **a2.** Coronal sections showing the channelrhoposin/EYFP+ (green), cell-type specific expression pattern in rCA2 in an Amigo2-iERT2-cre+ mouse (scale bar = 100 µm). **b1-b4.** EYFP+ axons originating from rCA2 neurons were present in the more dorsal and intermediate extents of caudal hippocampus. The EYFP+ axons were largely absent from the more ventral regions of the cCA1 region (scale bars = 100 µm).


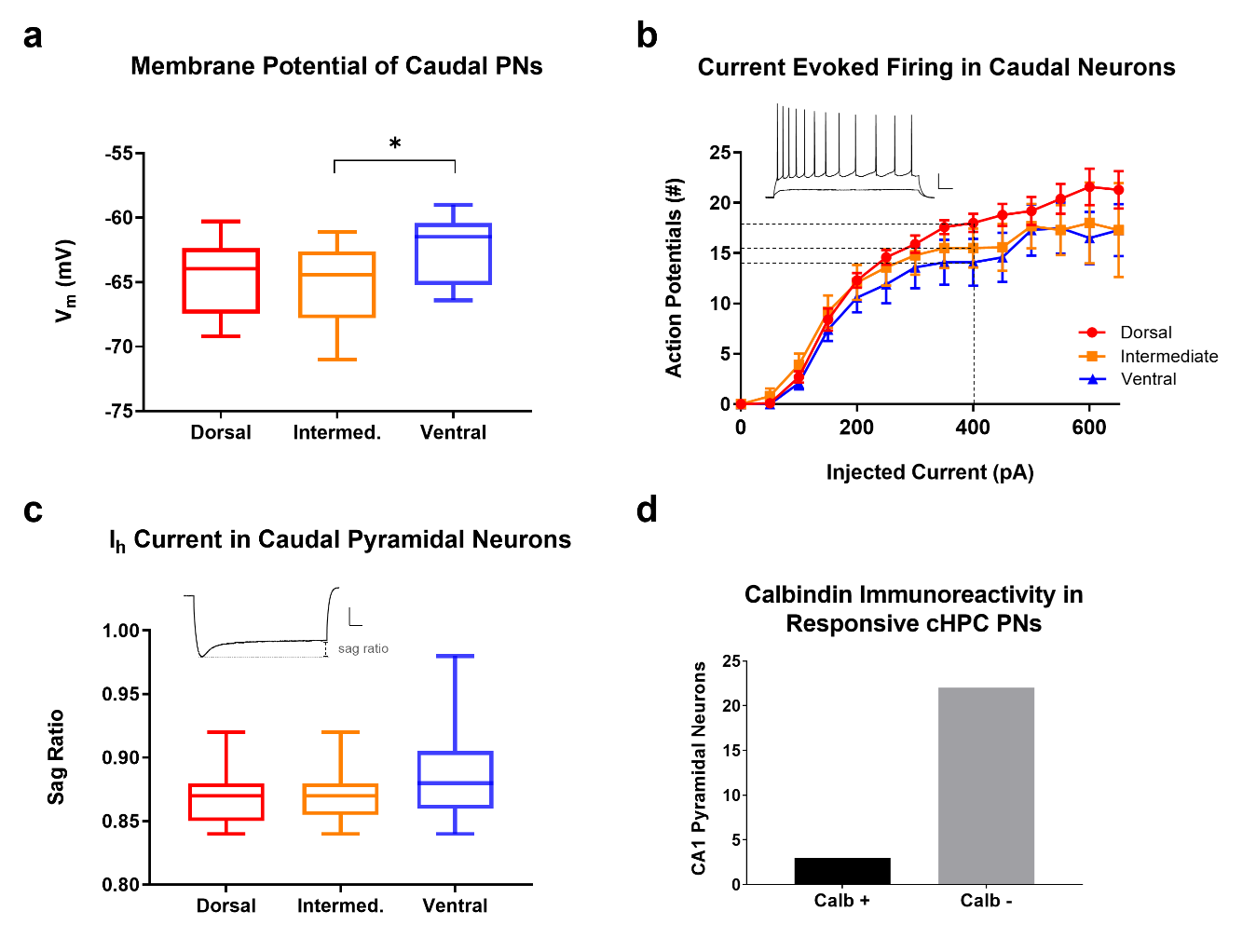


**Supplementary Figure 5:** Intrinsic properties of CA1 projection neurons are similar along the D-V axis of the caudal hippocampus (cHPC).

**a.** Min/max plots showing the intrinsic membrane potential (V_m_) of cCA1 pyramidal neurons. The average V_m_ of dorsal and intermediate CA1 pyramidal neurons were not significantly different, while intermediate CA1 neurons had a significantly more hyperpolarized membrane potential than ventral cHPC neurons Dorsal: -64.58 ± 0.73 mV vs. Intermediate: -64.95 ± 0.53 mV vs. Ventral: -62.43 ± 0.72 mV, n=16, 31 and 12 cells, from 11, 17, and 7 mice, respectively, one-way ANOVA F=3.468 p=0.038; Dorsal vs. Intermediate, Holm-Sidak *p*=0.67; Dorsal vs. Ventral, Holm-Sidak *p*=0.11; Intermediate vs. Ventral, Holm-Sidak *p*=0.0.035). **b.** cCA1 neurons exhibited similar input/output curves in response to 500 ms steps of depolarizing current (action potentials (APs) elicited with 400 pA current injections; Dorsal: 18.0 ± 0.91 APs vs. Intermediate: 15.5 ± 1.95 APs vs. Ventral: 15.4 ± 2.32 APs, 9 cells from 6 mice, 8 cells from 8 mice, 8 cells from 7 mice, respectively, scale bars = 20 mV x 100 ms). **c.** The voltage sag ratio following a 500 ms hyperpolarizing current injection, a measure of Ih current activation, did not significantly differ in CA1 neurons along the dorsal-intermediate-ventral axis of cHPC (Dorsal: 0.87 ± 0.01 vs. Intermediate: 0.87 ± 0.01 vs. Ventral: 0.89 ± 0.01, n=15, 17 and 9 cells, respectively, scale bars = 20 mV x 100 ms)). **d.** Neurons that received excitatory synaptic input from rostral CA2 are predominately negative for calbindin (Calbindin+ = 3 cells vs. Calbindin- = 22 cells). * *p<*0.05.


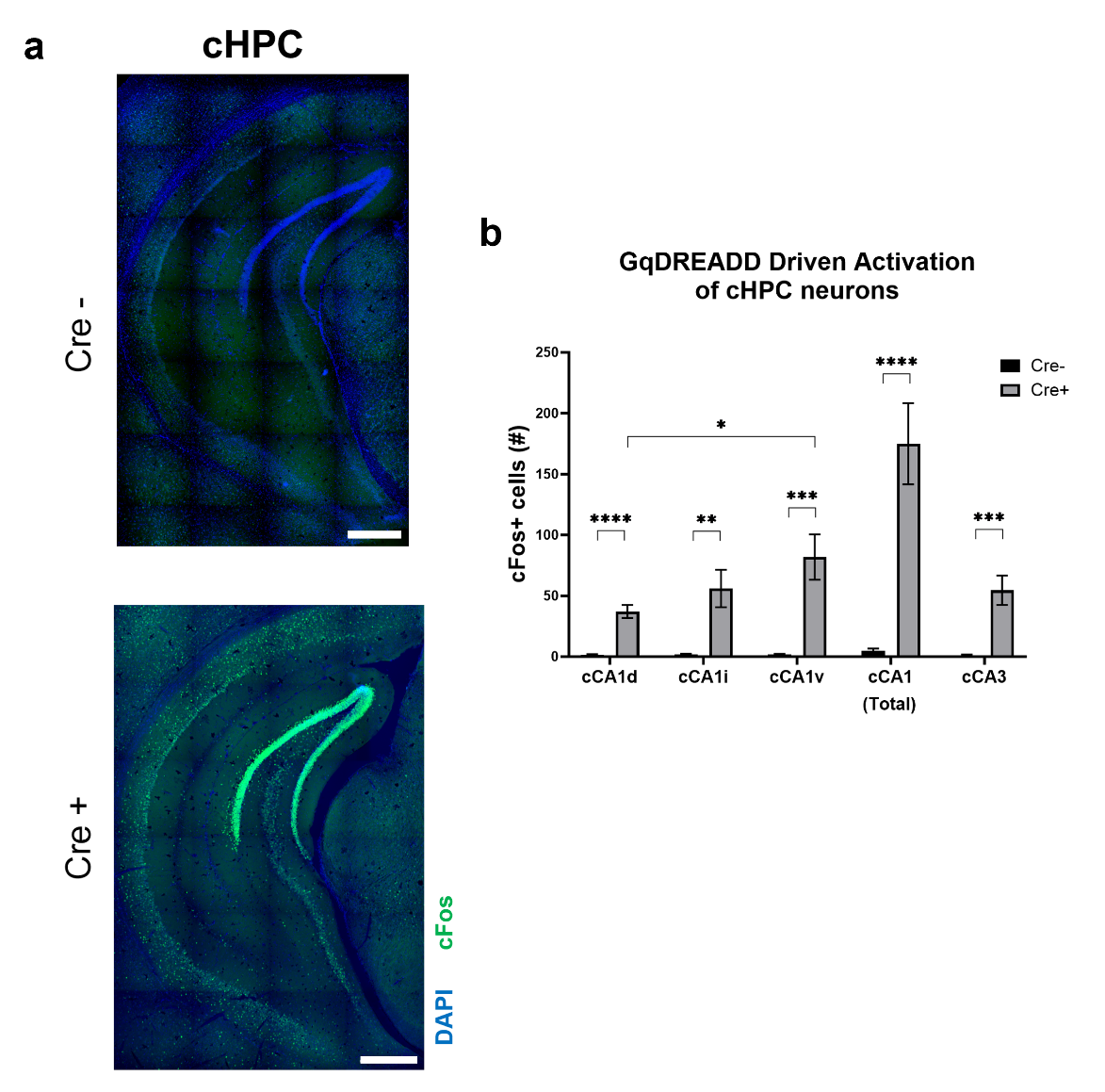


**Supplementary Figure 6**: Gq-DREADD mediated activation of CA2 neurons modulates cFos levels in the caudal hippocampus.

a. Coronal sections from the caudal hippocampus of cre- (top) and cre+ (bottom) Amigo2-icreERT2 mice immunohistochemically stained for the IEG cFos (scale bars = 500 µm). b**.** *in vivo* activation of area CA2 via bilateral expression of Gq-DREADDs causes a significant increase in cFos expression along the entirety of the caudal CA1 region of cre+ mice compared to cre- mice (cHPC: two-way ANOVA, between groups F= 6.62, p<0.0001, within groups F=64.46, p<0.0001) (cCA1 total: cre-: 4.9 ± 1.77 cFos+ cells vs. cre+: 175.00 ± 33.4 cFos+ cells, 10 hemispheres from 5 animals and 10 hemispheres from 6 animals, respectively, unpaired *t-test* p<0.0001). The significant increase in cFos+ staining is observable within the dorsal, intermediate, and ventral cCA1 subregions (cCA1d: cre-: 1.5 ± 0.6 cFos+ cells vs. cre+: 37.2 ± 5.4 cFos+ cells, p<0.0001 :: cCA1i: cre-: 1.7 ± 0.8 vs. cre+: 50.0 ± 15.4, p=0.0024 :: cCA1v: cre-: 1.7 ± 0.6 vs. cre+: 81.9 ± 18.6, p=0.0004), as well as, with area CA3 (cCA3: cre-: 1.2 ± 0.7 vs. cre+: 54.6 ± 12.1, p=0.0003). Within the cre+ group, following activation of CA2 neurons, the ventral subregion of cCA1 exhibited significantly higher levels of cFos staining when compared to more dorsal regions (cCA1v: 81.9 ±18.6 vs. cCA1d: 37.2 ± 5.4 cFos+ cells, p=0.03). * *p<*0.05, ** *p*<0.01, *** *p*<0.001, **** *p*<0.0001.
